# Piezo1 in hypertrophic chondrocytes regulates osteoclastogenesis in endochondral ossification

**DOI:** 10.7150/ijbs.126065

**Published:** 2026-04-23

**Authors:** Miriam E.A. Tschaffon-Müller, Astrid Schoppa, Franziska Eckl, Laura J. Brylka, Melanie Haffner-Luntzer, Timur A. Yorgan, Sandra Dieterich, Christoph Kölbl, Michael Amling, Thorsten Schinke, Anita Ignatius

**Affiliations:** 1 Institute of Orthopaedic Research and Biomechanics, University Medical Center Ulm, Ulm, Germany.; 2 Department of Osteology and Biomechanics, University Medical Center Hamburg-Eppendorf, Hamburg, Germany.

**Keywords:** Piezo1, bone homeostasis, fracture healing, osteoclastogenesis

## Abstract

Ion channels of the Piezo family are key mechanosensors in diverse tissues, including bone. Recent studies have demonstrated that Piezo1 in differentiating chondrocytes is critical for endochondral ossification during bone development and regeneration. During endochondral ossification, chondrocytes undergo hypertrophy prior to apoptosis or transdifferentiation into bone-forming osteoblasts, thereby significantly contributing to new bone formation. Here, we investigated the specific role of Piezo1 in hypertrophic chondrocytes using a mouse model with conditional *Piezo1* deficiency under control of the collagen X promoter (*Piezo1^Col10a1-Cre^*). These mice exhibited a pronounced osteopenic bone phenotype and impaired callus maturation. Notably, hypertrophic chondrocyte apoptosis and chondrocyte-to-osteoblast transdifferentiation remained unaffected during endochondral ossification in *Piezo1^Col10a1-Cre^* mice. Instead, these mice displayed markedly increased osteoclast numbers in the primary spongiosa beneath the growth plates and within the fracture callus. Further *in vivo* and *in vitro* analysis revealed that Piezo1 regulates osteoclastogenesis by repressing receptor activator of NF-κB ligand and inducing osteoprotegerin expression in hypertrophic chondrocytes. Collectively, our findings identify Piezo1 as an important regulator of hypertrophic chondrocyte-osteoclast communication during endochondral bone formation.

## Introduction

Bone development, growth, and remodeling are highly complex processes involving multiple cell types with distinct functions. These include: bone-forming osteoblasts and chondrocytes, both derived from mesenchymal precursor cells; osteocytes, which are terminally differentiated osteoblasts embedded within the mineralized bone matrix; and bone-resorbing osteoclasts which are of hematopoietic origin. The function and interaction of these cells are regulated by multiple mechanisms, including mechanical stimuli 1, 2. Most of the skeleton develops via endochondral ossification, a process in which a cartilage template is initially formed and later replaced by bone tissue 3. Few bones, such as the flat bones of the skull, form through intramembranous ossification, in which mesenchymal progenitors directly differentiate into osteoblasts and form bone directly without a cartilage template. Postnatally, longitudinal bone growth occurs at the growth plate, a cartilaginous structure beneath the bone epiphysis. Within the growth plate, chondrocytes are organized in columns and undergo a sequence of proliferation and differentiation into pre-hypertrophic and subsequently hypertrophic chondrocytes 4. Hypertrophic chondrocytes are characterized by an enlarged cell volume and type X collagen (*Col10a1*) expression 4, 5. At the cartilage-to-bone transition zone, terminally differentiated hypertrophic chondrocytes either undergo apoptosis 6, 7 or transdifferentiate into osteoblasts which contribute to new bone formation in the primary spongiosa 8, 9. This structure is subsequently replaced by the secondary spongiosa through recruitment of osteoclast and osteoblast precursor cells. Throughout life, bone is continuously remodeled through the coordinated actions of osteoblasts and osteoclasts. Key regulators of osteoblast-osteoclast communication include the cytokine receptor activator of NF-κB ligand (RANKL), which promotes bone resorption, and its decoy receptor osteoprotegerin (OPG), which inhibits RANKL-induced osteoclastogenesis 10, 11. This dynamic turnover ensures bone renewal, repair, and adaptation to mechanical demands 1, 12. Following fracture, bone healing recapitulates processes of both intramembranous and endochondral bone formation that underlie skeletal development and remodeling 13, 14.

Mechanical loading is a crucial regulator of bone formation during development, postnatal growth, and fracture healing, and is also central in bone remodeling 15, 16. Various skeletal cells, including mesenchymal progenitor cells, osteoblasts, osteocytes, and chondrocytes, sense and respond to mechanical stimuli via multiple pathways. These pathways involve, for example, primary cilia, integrins and ion channels 17, 18. Among these channels, the mechanically activated Piezo ion channels (Piezo1 and Piezo2) have attracted considerable attention in recent years 19-21. In bone, Piezo1 is a crucial regulator of osteoanabolic processes. Several groups, including ours, have demonstrated that Piezo1 is essential for mechanosensation at various stages of osteoblast differentiation, mediating the anabolic effects of mechanical loading 22-25. Furthermore, we showed that the targeted deletion of Piezo1 in osteoblasts (*Piezo^Runx2-Cre^* mice) induces severe osteoporosis, primarily affecting the trabecular bone beneath the chondrogenic growth plate 22. On the basis of these findings, we hypothesized that Piezo1 also plays a key role in growth plate chondrocytes during endochondral ossification, because Runt-related transcription factor 2 (Runx2), which encodes a transcription factor critical for osteoblast differentiation, is not only expressed in osteoblast progenitor cells but also in pre-hypertrophic chondrocytes 22. To test this, we previously generated mice with Piezo1 deletion in chondrocytes during differentation (*Col2a1-Cre*). These mice exhibited a nearly-complete loss of trabecular bone beneath the growth plate, whereas cortical bone remained intact 22, 26. Additionally, not only was articular cartilage degeneration attenuated, but also osteophyte formation was less pronounced in *Piezo1^Col2a1-Cre^* mice in models of surgically induced and age-related osteoarthritis. We identified *Ccn2*, encoding connective tissue growth factor, and *Ptgs2*, encoding cyclooxygenase 2, as potentially relevant downstream targets of Piezo1 in chondrocytes 26. Other studies showed that *Piezo1^Col2a1-Cre^* mice exhibited impaired fracture healing, characterized by defective cartilage-to-bone transition in the fracture callus. This was associated with the upregulation of apolipoprotein E (ApoE) and enhanced chondrocyte senescence 27, as well as defective beta-catenin signaling, most likely regulated by Lars2 28. Another study identified the Sirt1-Piezo1 axis as an important regulator of endochondral ossification 29. Collectively, these findings established Piezo1 as a crucial regulator of chondrocyte function in both physiologic and pathologic endochondral ossification processes, although the underlying molecular mechanisms remain incompletely defined.

In the present study, we generated *Piezo1^Col10a1-Cre^* mice to specifically investigate the role of Piezo1 in late-stage hypertrophic chondrocytes during endochondral bone formation. Our findings from comprehensive skeletal phenotyping and fracture healing analyses indicated that Piezo1 in hypertrophic chondrocytes does not affect chondrocyte-to-osteoblast transdifferentiation or chondrocyte apoptosis but is crucial for their communication with bone-resorbing osteoclasts. Mechanistically, Piezo1 regulates RANKL and OPG expression in hypertrophic chondrocytes, thereby promoting endochondral ossification by modulating osteoclast formation and activity.

## Materials and Methods

### Mouse model

To generate mice with a specific knockout of *Piezo1* in hypertrophic chondrocytes (referred as *Piezo1^Col10a1-Cre^*), *Piezo1l^fox/flox^* mice were crossed with Col10a1-Cre mice. Cre^-^ littermates (referred to as *Piezo1^fl^*) were used as controls. *Piezo1l^fox/flox^* mice 30 were crossed with Col10a1-Cre mice 31 as previously described. Genotyping was performed using the following primers: *Piezo1-flox forward: GGA GGG TTG CCT TGT T, Piezo1-flox reverse: ACT CAT CTG GGG TGA G; Col10a1-Cre forward: TTT AGA GCA TTA TTT CAA GGC AGT TTC CA, Col10a1-Cre reverse 1: AGG CAA ATT TTG GTG TAC GG, Col10a1-Cre reverse 2: ATC ATT CCG CTG TAC TAG TAG CTC AAG CCA ATC.*

### Animal studies

Mice were housed in groups of 1-5 per cage in standard polycarbonate cages (size 16 × 22 × 14 cm) under standard laboratory conditions (22°C, 60% humidity, 12 h light/ 12 h dark cycle). Animals had *ad libitum* access to tap water and a standard mouse diet (SNIFF V1534). Animals were randomly assigned to the different groups according to their genotype and sex. All analyses were performed in a blinded manner. Baseline skeletal phenotyping was performed in both male and female mice to capture potential sex-specific effects. Fracture healing experiments were performed in female mice only to reduce mouse numbers and account for 3R principles, as both male and female mice showed a bone phenotype.

### Whole-body X-ray imaging

Euthanized mice were stretched for 20 min, after which organs and skin were removed. The bodies were subsequently imaged using an MX20 X-ray device (Faxitron X-Ray Corp., Buffalo Grove, IL, USA) operated at 35 kV with a 5-second exposure time.

### Femur osteotomy

At 12 weeks of age, female *Piezo1^fl^* and *Piezo1^Col10a1-Cre^* mice underwent a standardized osteotomy of the right femur, stabilized with a semi-rigid external fixator, as described previously 32. Anesthesia was induced and maintained with 2% isoflurane. Analgesia was provided by administering tramadol (0.1 mg/mL) in the drinking water from 1 day before until 3 days after surgery, together with an additional injection of tramadol (25 mg/kg body weight) 30 minutes prior to fracture surgery. Mice were euthanized 10, 14 or 21 days post-fracture.

### Three-point bending test

Biomechanical testing of intact and fractured femora from mice euthanized 21 days post-fracture was performed using a nondestructive three-point bending test, as described previously 32. Briefly, a maximum axial load of up to 2 N was applied to the cranio-lateral callus side, while a load of 4 N was applied to the cranio-lateral side of the contralateral femur using a material testing machine (1454, Zwick, Ulm, Germany). The bending stiffness was calculated using the slope of the load-deflection curve. The relative flexural rigidity of the fractured femur was calculated as the ratio to that of the contralateral femur. As the bending stiffness reflects “apparent Young's modulus x moment of inertia”, we further calculated the apparent Young's modulus as an important parameter for callus biomechanical material quality.

### µCT analysis

Left femora from unfractured mice and right femora from mice euthanized 21 days post-fracture were fixed in formalin for 48 h and scanned using a Skyscan 1172 µCT system (Bruker, Billerica, MA, USA) operating at 50 kV, 200 µA and with an isotrophic voxel resolution of 8 μm. Three-dimensional analyses of the µCT scans were performed using CTAn software (Bruker) according to the ASBMR guidelines 33. For unfractured femora, the volume of interest (VOI) for trabecular bone was defined as a 280-μm-thick region between the cortices located 360 μm away from the growth plate, while the VOI for cortical bone was defined as a 160 μm thick region surrounding the cortex distal to the third trochanter. The VOI for vertebrae was defined as a cylindrical region of 800 µm diameter at the center of lumbar vertebra L4. For fractured femora, the VOI encompassed the entire periosteal callus between the two inner pinholes. TMD was calibrated using two phantoms with defined hydroxyapatite (HA) contents of 250 and 750 mg HA/cm^3^. The thresholds for mineralized bone tissue were set at 394 mg HA/cm^3^ for trabecular bone and at 642 mg HA/cm^3^ for unfractured cortical bone and the fracture callus. Three-dimensional reconstructions of scanned bones were generated using CT Vol and CT Vox software (Bruker).

### Histology and histomorphometric analysis

Rib sections were embedded in methyl methacrylate without decalcification and stained with v. Kossa. Femora were subjected to decalcified paraffin histology as described previously 34. Sections of unfractured femora were stained with Toluidine blue and for TRAP. Fracture callus sections were stained with Safranin O and for TRAP. Osteoblasts and osteoclasts were analyzed using Osteomeasure software (Decatur, Georgia, USA). Fracture callus tissue composition was determined on Safranin-O-stained sections using Leica Application Suite X software (Leica, Wetzlar, Germany).

### Immunohistochemistry

Paraffin sections of femora were stained for Sox2, Runx2, RANKL, OPG, and Piezo1 using the following antibodies, incubated overnight at 4°C: rabbit anti-mouse Sox2 (1:100; #ab97959, Abcam), rabbit anti-mouse Runx2 (1:50; #8486, Cell Signaling), rabbit anti-RANKL (1:50; #23408-1-AP, Thermo Fisher Scientific), rabbit anti-osteoprotegerin (1:50; #ab183910, abcam), and rabbit anti-Piezo1 extracellular domain (1:50; #82625-4-RR, Proteintech). Antigen retrieval was performed for 20 min at 95°C using citrate buffer (for Sox2, Runx2, RANKL, OPG) or Tris-EDTA (for Piezo1), followed by blocking with 10% goat serum. Sections were subsequently incubated with biotin-XX-goat anti-rabbit secondary antibody (1:200, #B2770, Life technologies, Carlsbad, CA, USA) for 1 h at room temperature, followed by a 30 min incubation with horseradish peroxidase-conjugated streptavidin (#PK-6100, VECTASTAIN Elite ABC-HRP Kit, Vector Laboratories, Burlingame, UK) and a 2-5 min incubation with NovaRED substrate (#SK-4800, Vector NovaRED Substrate Kit, Vector Laboratories). Counterstaining was performed with hematoxylin (1:2000, Waldeck, Münster, Germany). Sections were analyzed using Osteomeasure and Leica Application Suite X software. Negative control stainings were performed with species-specific IgG control antibodies (supplemental figure S6).

### TUNEL assay

Chondrocyte apoptosis was assessed using the CFTM 488A TUNEL Apoptosis Detection Kit (Biotium) following the manufacturer's instructions. Briefly, deparaffinized sections were permeabilized in 0.2% Triton X-100 for 30 min, washed twice with PBS, and incubated in TUNEL equilibration buffer for 5 min. The TUNEL reaction was performed for 1 h at 37 °C, followed by washing with PBS containing 0.1% Triton X-100 and 5 mg/mL bovine serum albumin. Sections were subsequently counterstained with Hoechst 33258 (Sigma-Aldrich).

### Cell culture

Murine chondrogenic ATDC5 cells (2500 cells/cm^2^) were cultured in chondrogenic differentiation medium (DMEM/F12 (1:1) (Gibco), supplemented with 5% fetal calf serum (FCS) (Merck Millipore), 1% L-glutamine (Gibco), 1% penicillin/ streptomycin (Gibco), human transferrin (10 μg/ml, Sigma), sodium selenite (30 nM), human insulin (10μg/ml, Sigma), and ascorbate 2-phosphate (0.2 mM, Sigma). Cells were maintained under hypoxic conditions (6% O_2_) for 7 or 10 days. Yoda1 (5 µM; Tocris Bioscience, Bristol, UK) or GsMTx4 (4 µM; abcam, Cambridge, UK) was added to the medium throughout the entire differentiation period. Furthermore, a standardized *in vitro* assay for chondrocyte-osteoblast transdifferentiation using the ATDC5 chondrogenic cell line was performed as described previously 35. Briefly, cells were incubated for 7 days in chondrogenic differentiation medium at 6% oxygen level, followed by 2 days of incubation in osteogenic differentiation medium at physiological oxygen concentration.

Human chondrogenic C28/I2 cells (4000 cells/cm^2^) were cultured in DMEM/F12 (1:1) (Gibco), supplemented with 10% FCS (Merck Millipore), 1% penicillin/ streptomycin (Gibco), and 100 ng/mL recombinant human BMP-2 (Thermo Fisher Scientific) under hypoxic conditions (6% O_2_) for 7 or 10 days. Yoda1 or GsMTx4 was added at the concentrations described above. Vehicle solution was the respective amount of DMSO for Yoda1 and water for GsMTx4. In an additional experiment, N-[N-(3,5-Difluorphenacetyl)-L-alanyl]-S-phenylglycine-tert-butyl ester (DAPT) was added to the medium of Yoda1-treated cells at a concentration of 50 µM for the entire chondrogenic differentiation period. siRNA-mediated knockdown of Notch3 was achieved by using the silencer-select Notch3 siRNA (#155632, ThermoFisher) according to a previously published protocol 34. A non-targeting siRNA (siNT) was used a control.

### Gene expression analysis

Total RNA was isolated from cultured ATDC5 cells and C28/I2 cells using the RNeasy Mini Kit (Qiagen), and each sample was treated with DNase (Qiagen). cDNA was synthesized using Omniscript Reverse Transcriptase (Qiagen) and semi-quantitative real-time PCR was performed using Platinum™ SYBR™ Green qPCR SuperMix-UDG (Thermo Fisher Scientific). Relative gene expression levels were normalized to *GAPDH* and calculated using the ΔΔCT method. Primer sequences are provided in Table S1.

### Statistics

Statistical analyses were performed using GraphPad Prism 9 (GraphPad Software, San Diego, CA, USA). Data distribution was assessed for normality using the Shapiro-Wilk test. For comparisons between two groups, an unpaired two-tailed Student's t-test was used for normally distributed data, whereas the non-parametric Mann-Whitney U test was applied when normality was not met. For comparisons involving more than two groups or multiple factors, two-way ANOVA was performed, followed by Bonferroni's post hoc multiple comparisons test where appropriate.

Data are presented as box plots showing the median, interquartile range, and minimum to maximum values, with individual data points displayed. The number of biological replicates (n) is indicated in the respective figure legends. Differences were considered statistically significant at p < 0.05, and exact p-values are reported where needed to see trends.

## Results

To analyze the function of Piezo1 in late-stage hypertrophic chondrocytes, we generated mice with conditional inactivation of Piezo1 (*Piezo1^Col10a1-Cre^*) and characterized their skeletal phenotype. Genotyping of ear punches confirmed that *Piezo1^Col10a1-Cre^* mice, but not Cre-negative littermate controls (*Piezo1^fl^*) exhibited a *Col10a1-Cre* PCR product Fig. S1 A). Wildtype *Piezo1^+/+^* displayed no floxed *Piezo1* PCR product, whereas *Piezo1^fl^* mice lacked the wildtype Piezo1 PCR band (Fig. S1 B). Immunohistochemical staining verified the absence of the Piezo protein in hypertrophic chondrocytes of *Piezo1^Col10a1-Cre^* mice (Fig. S1 C).

**Figure 1:**
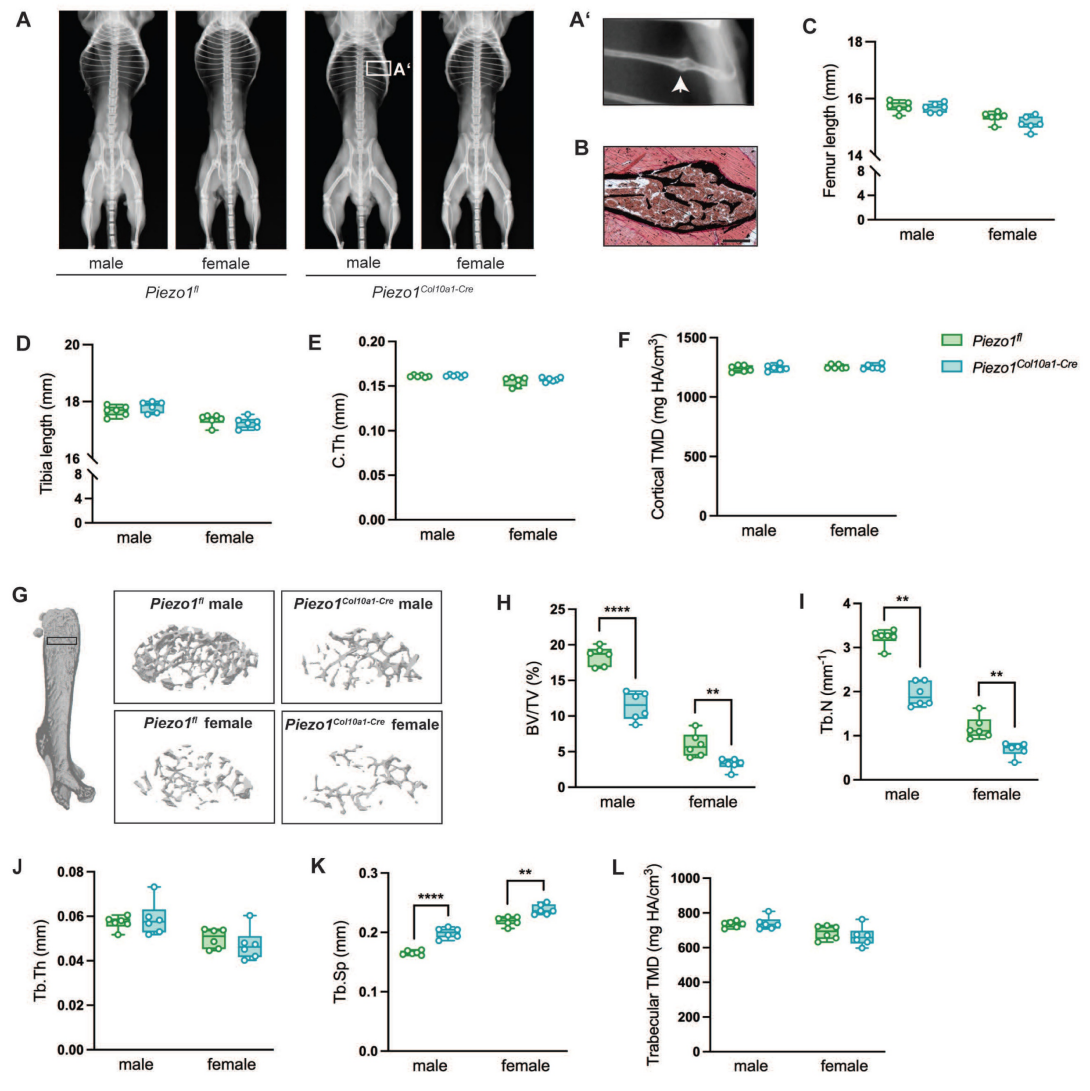
(A) Whole-body X-ray images of male and female *Piezo1^fl^* and *Piezo1^Col10a1-Cre^* mice, (A') higher magnification of a rib fracture of a male *Piezo1^Col10a1-Cre^* mouse (white arrow) and (B) v. Kossa-stained section of the rib fracture. Scale bar represents 200 µm. (C) Femur and (D) tibia lengths of *Piezo1^fl^* and *Piezo1^Col10a1-Cre^* mice, (E) cortical thickness (C.Th) and (F) cortical tissue mineral density (TMD) in femora. (G) Representative images of analyzed volumes of interest (VOIs) of femora and (H) bone volume/tissue volume ratio (BV/TV), (I) trabecular number (Tb.N), (J) trabecular thickness (Tb.Th), (K) trabecular separation (Tb.Sp), and (L) trabecular TMD in femora. N = 6, **p<0.01, ****p<0.0001. Box plots are depicted as interquartile range (=box), median (=line) and minimum to maximum values (=whiskers). The volume of interest (VOI) for trabecular bone was defined as a 280-μm-thick region between the cortices located 360 μm away from the growth plate, while the VOI for cortical bone was defined as a 160 μm thick region surrounding the cortex distal to the third trochanter.

At 12 weeks of age, male and female *Piezo1^Col10a1-Cre^* mice displayed no overt skeletal abnormalities compared to *Piezo1^fl/fl^* controls (Fig. 1 A), except for spontaneous rib fractures observed in two male *Piezo1^Col10a1-Cre^* mice (Fig. 1 A, B). Long bone length and cortical bone parameters were unaffected (Fig. 1 C-F). However, micro-computed tomography (µCT) analyses revealed markedly reduced trabecular bone mass in the secondary spongiosa of femora (Fig. 1 G-L) and in the spine (Fig. S2 A-F), as evidenced by a significantly decreased bone volume/tissue volume (BV/TV; Fig. 1 G, H; Fig. S2 A, B), reduced trabecular number (Tb.N; Fig. 1 I; Fig. S2 D), and increased trabecular separation (Tb.Sp, Fig. 1 K; Fig. S2 F).

**Figure 2:**
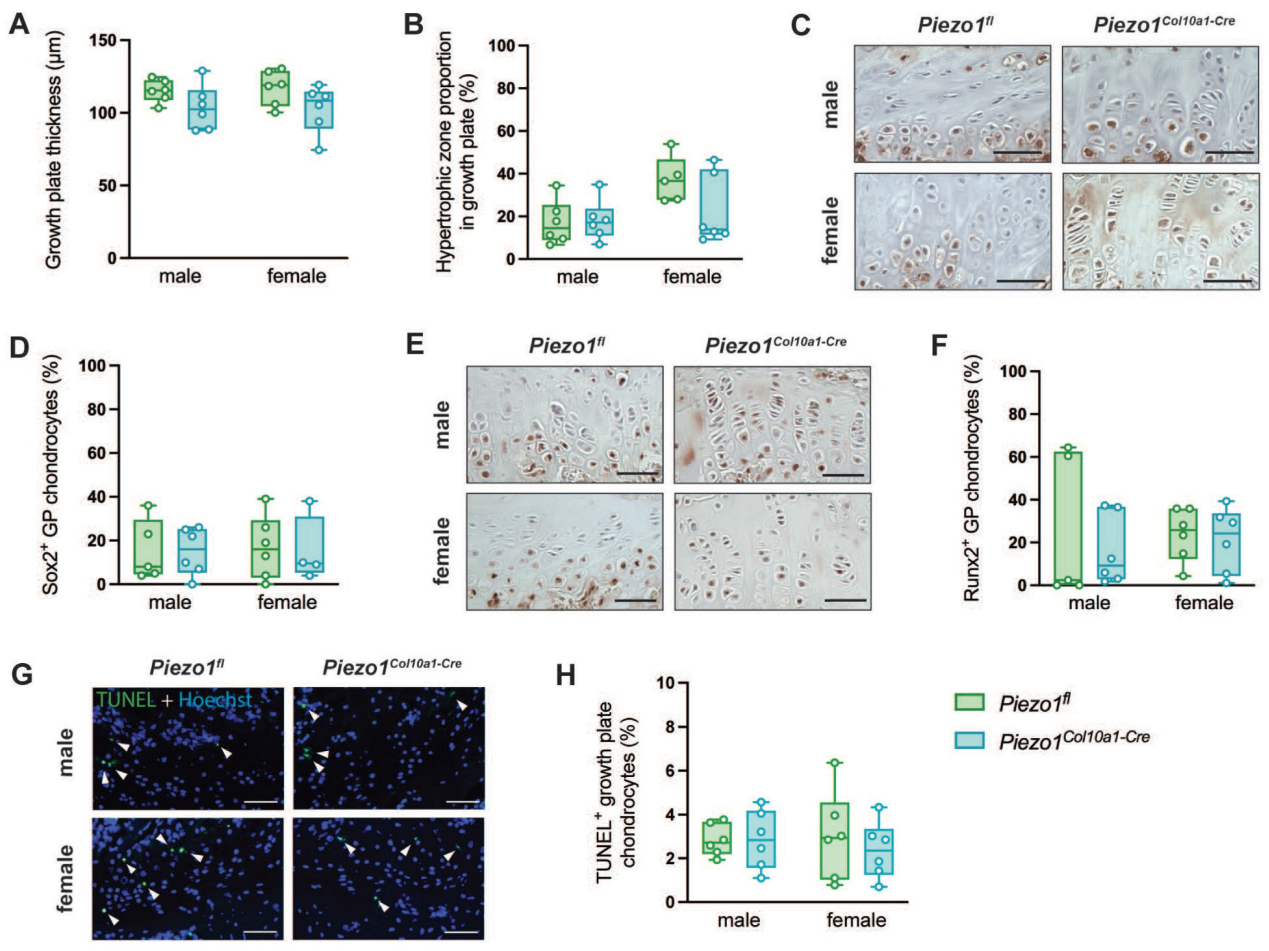
(A) Growth plate thickness and (B) proportion of the hypertrophic zone of the growth plate of *Piezo1^fl^* and *Piezo1^Col10a1-Cre^* mice. (C) Immunohistochemical staining of Sox2, (D) percentage of Sox2^+^ growth plate (GP) chondrocytes, (E) immunohistochemical staining of Runx2, and (F) percentage of Runx2^+^ GP chondrocytes in 12-week-old male and female *Piezo1^fl^* and *Piezo1^Col10a1-Cre^* mice. (G) TUNEL staining with TUNEL^+^ chondrocytes marked by arrowheads and (H) percentage of TUNEL^+^ growth plate chondrocytes in 12-week-old male and female *Piezo1^fl^* and *Piezo1^Col10a1-Cre^* mice. N = 5-6. Scale bars represent 50 µm. Box plots are depicted as interquartile range (=box), median (=line) and minimum to maximum values (=whiskers).

**Figure 3:**
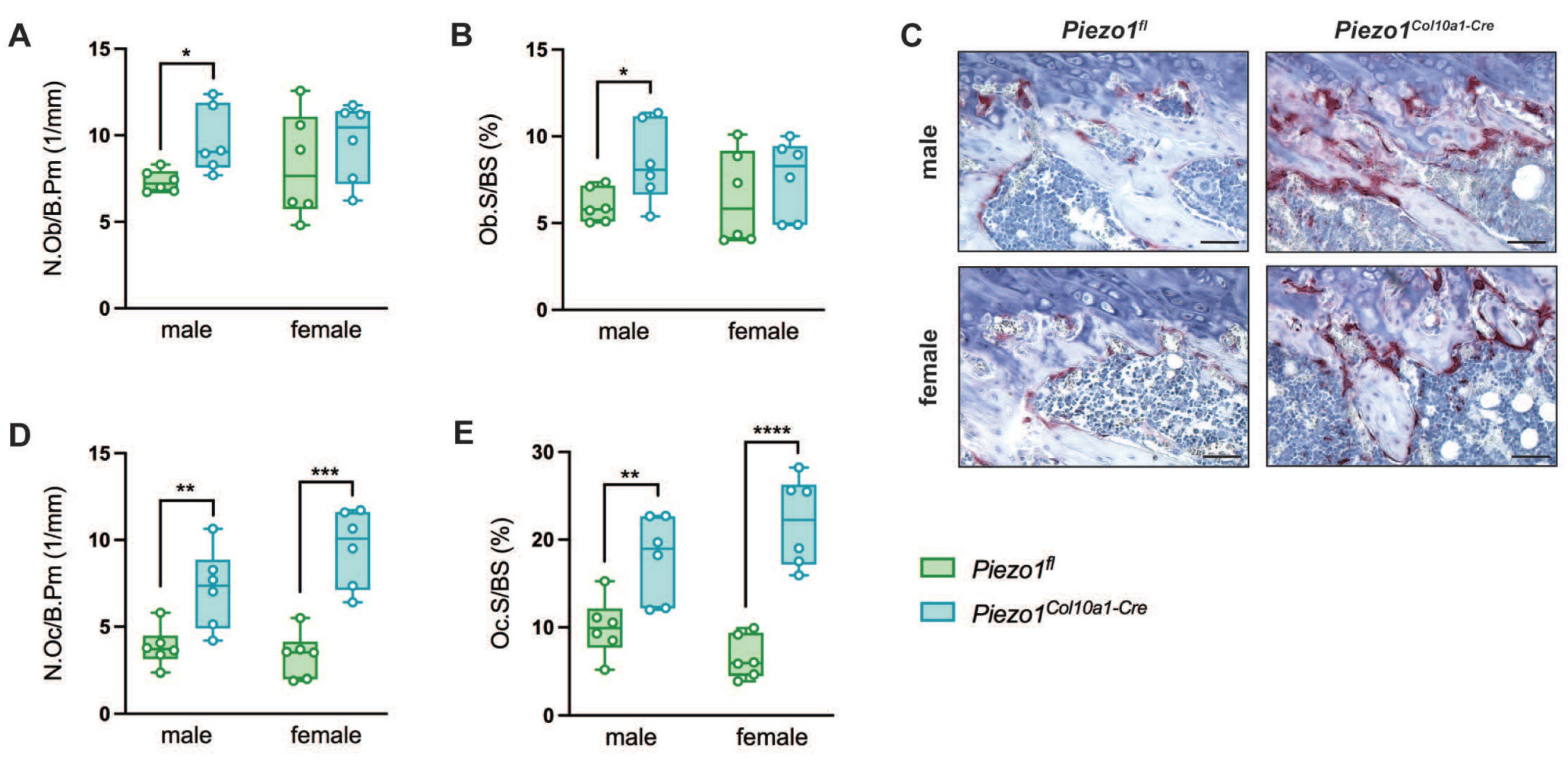
(A) number of osteoblasts/bone perimeter (N.Ob/B.Pm), (B) osteoblast surface/bone surface (Ob.S/BS), (C) representative images of the primary spongiosa of TRAP-stained femur sections, (D) number of osteoclasts/bone perimeter (N.Oc/B.Pm) and (E) osteoclast surface/bone surface (Oc.S/BS) in the primary spongiosa of femora of 12-week-old male and female *Piezo1^fl^* and *Piezo1^Col10a1-Cre^* mice. N = 6, *p<0.05, **p<0.01, ***p<0.001, ****p<0.0001. Scale bars represent 50 µm. Box plots are depicted as interquartile range (=box), median (=line) and minimum to maximum values (=whiskers). N=6.

**Figure 4:**
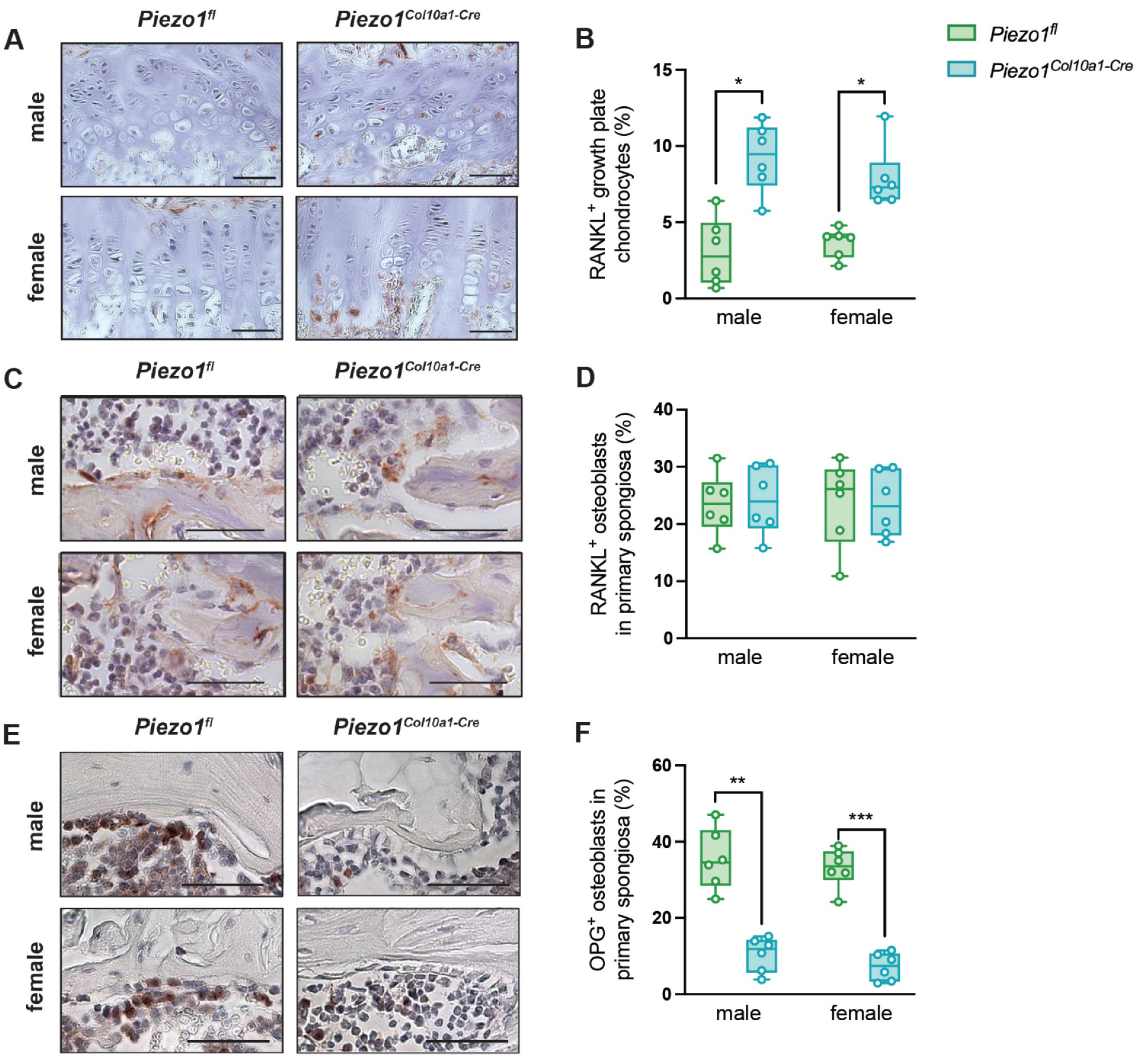
(A) Immunohistochemical staining of RANKL, (B) percentage of RANKL^+^ growth plate (GP) chondrocytes, and C), D) RANKL^+^ osteoblasts in the primary spongiosa. (E) Immunohistochemical staining of OPG and (F) percentage of OPG^+^ osteoblasts in the primary spongiosa in 12-week-old male and female *Piezo1^fl^* and *Piezo1^Col10a1-Cre^* mice. N = 6, *p<0.05, **p<0.01, ***p<0.001. Scale bars represent 50 µm. Box plots are depicted as interquartile range (=box), median (=line) and minimum to maximum values (=whiskers). N=4-6.

To evaluate endochondral bone formation at the growth plate, we performed histomorphometric and immunohistochemical analyses. Neither growth plate thickness nor the proportion of the hypertrophic zone was altered in *Piezo1^Col10a1-Cre^* mice (Fig. 2 A, B). Because terminally differentiated chondrocytes either undergo apoptosis or transdifferentiate into osteoblasts, we assessed both processes. Expression of the pluripotency marker SRY-related HMG-box 2 (Sox2) (Fig. 2 C, D), shown previously to be associated with transdifferentiating chondrocytes (36, and the early osteoblast marker Runx2 (Fig. 2 E, F) remained unchanged in chondrocytes upon *Piezo1* deletion. TUNEL staining likewise revealed no differences in chondrocyte apoptosis (Fig. 2 G, H). In the primary spongiosa beneath the growth plate, osteoblast number (N.Ob/B.Pm) and surface (Ob.S/BS) were modestly increased in male *Piezo1^Col10a1-Cre^* mice but unaffected in females (Fig. 3 A, B), which might be due to different phases of endochondral ossification male and female mice are in at 12 weeks of age 37. By contrast, osteoclast number (N.Oc/B.Pm) and surface of (OcS/B.PS) were significantly increased in both sexes (Fig. 3 C-E). Further indicating increased bone resorption, both male and female* Piezo1^Col10a1-Cre^* mice exhibit significantly increased serum CTX levels (Table 1). To investigate mechanisms underlying the increased osteoclastogenesis, we performed immunohistochemical staining for RANKL and OPG. RANKL was markedly upregulated in hypertrophic chondrocytes of the growth plate of *Piezo1^Col10a1-Cre^* mice (Fig. 4 A, B), whereas OPG was not detected in these cells Fig. S3. By contrast, OPG expression in osteoblasts of the primary spongiosa was significantly lower in *Piezo1^Col10a1-Cre^* mice than in *Piezo1^fl/fl^* controls (Fig. 4 E, F), while this was not the case when looking at RANKL expression in osteoblasts of the primary spongiosa (Fig. 4 C, D).

**Figure 5:**
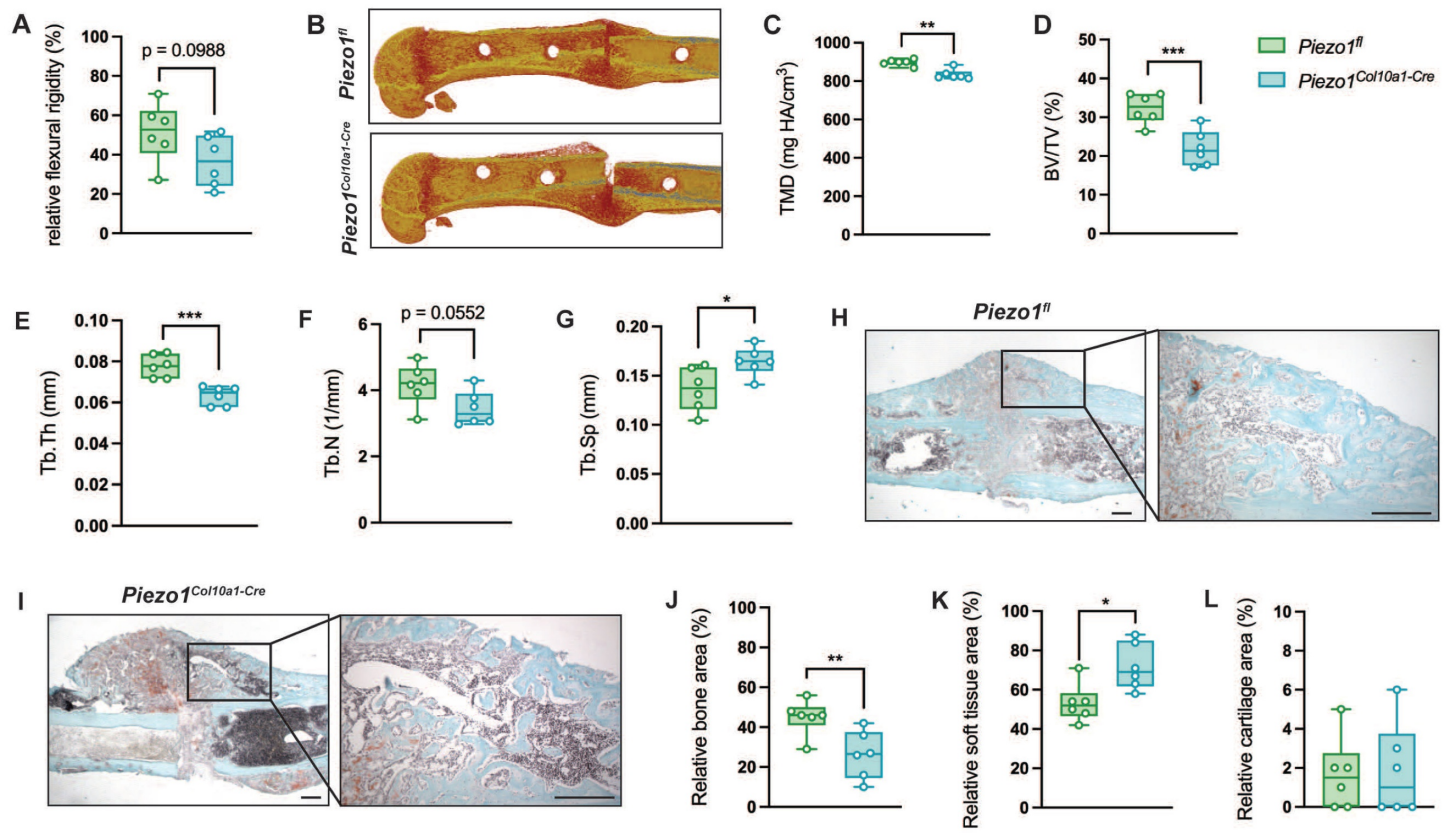
(A) Relative flexural rigidity of fractured femora, (B) representative 3D images of fracture calli, and (C) TMD, (D) BV/TV, (E) Tb.Th, (F) Tb.N and (G) Tb.Sp in the fracture callus of *Piezo1^fl^* and *Piezo1^Col10a1-Cre^* mice 21 days post-fracture. (H, I) Representative images of Safranin O-stained fracture callus sections (scale bars: 250 µm), and relative (J) bone (K) soft tissue, and (L) cartilage area in fracture calli 21 days post-fracture. N = 6, *p<0.05, **p<0.01, ***p<0.001. Box plots are depicted as interquartile range (=box), median (=line) and minimum to maximum values (=whiskers). N=6. The volume of interest encompassed the entire periosteal callus between the two inner pinholes.

We subsequently investigated the role of Piezo1 in hypertrophic chondrocytes during fracture healing, applying a standardized femur osteotomy to 12-week-old female *Piezo1^fl^* and *Piezo1^Col10a1-Cre^* mice. Callus maturation was impaired in *Piezo1^Col10a1-Cre^* mice. At 21 days post-fracture, fractured femora of *Piezo1^Col10a1-Cre^* mice displayed a non-significant trend towards reduced flexural rigidity (Fig. 5 A, p = 0.0988), while apparent Young's modulus was significantly reduced (*Piezo1^fl^*: 578.0 ± 245 vs. *Piezo1^Col10a1-Cre^*: 249.6 ± 47.2 N/mm^2^, p = 0.021054). µCT analyses of the fracture callus revealed diminished bone content and quality, as evidenced by significantly decreased tissue mineral density (TMD), BV/TV, Tb.Th, a non-significant trend toward reduced Tb.N (p = 0.0552) and significantly increased Tb.Sp (Fig. 5 B-G). Histomorphometry confirmed significantly reduced bone (Fig. 5 H-J) but showed increased soft tissue contents in the fracture callus (Fig. 5 K), whereas the cartilage content remained unaffected in *Piezo1^Col10a1-Cre^* (Fig. 5 L). At day 14, no differences were observed in relative bone Fig. S4 A), cartilage (Fig. S4 B) or soft tissue area (Fig. S4 C).

**Figure 6:**
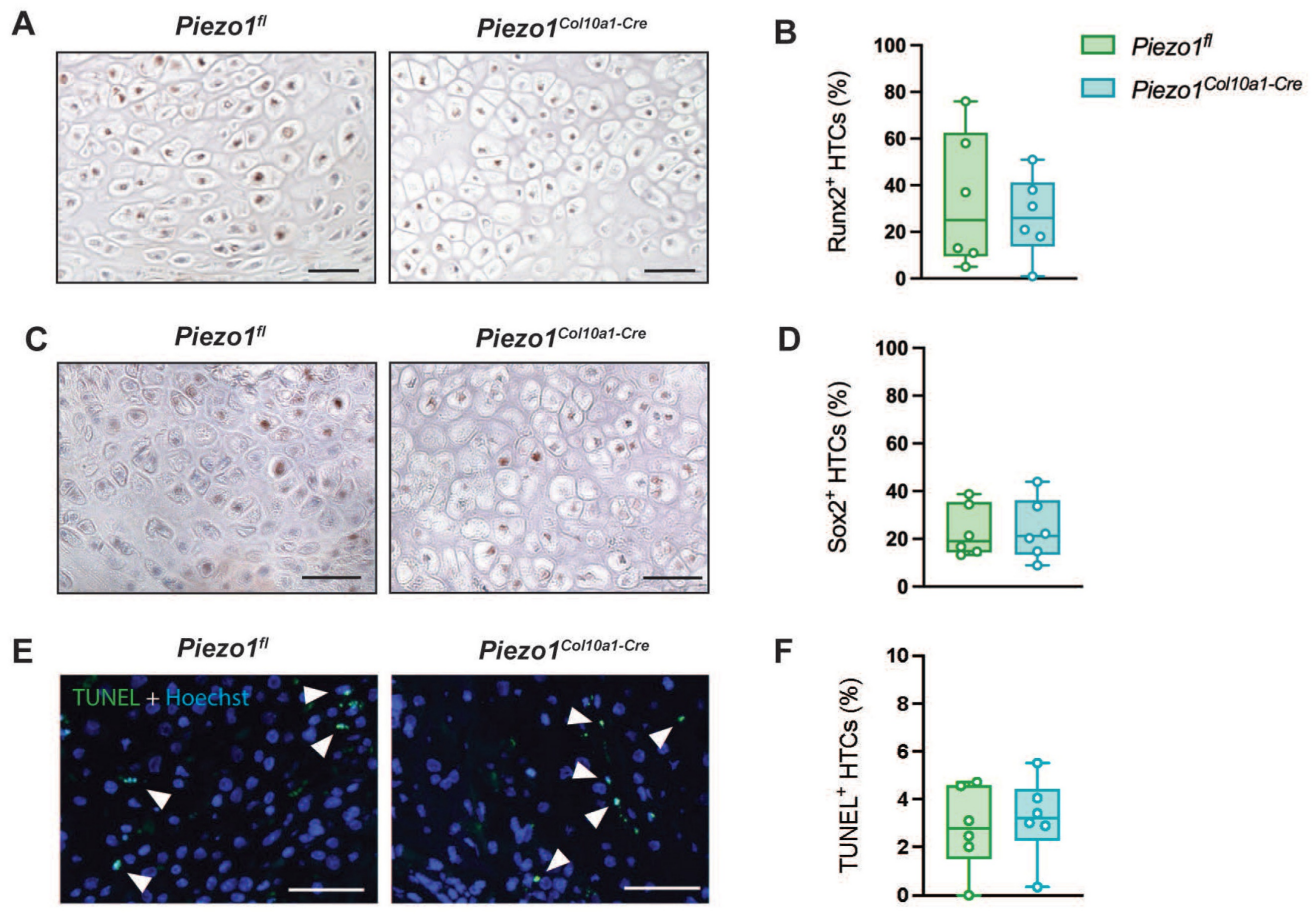
(A) Immunohistochemical staining of Runx2, (B) percentage of Runx2^+^ hypertrophic chondrocytes (HTCs), (C) immunohistochemical staining of Sox2, (D) percentage of Sox2^+^ HTCs, (E) TUNEL staining with TUNEL^+^ chondrocytes marked by arrowheads, and (F) percentage of TUNEL^+^ HTCs in the fracture callus of *Piezo1^fl^* and *Piezo1^Col10a1-Cre^* mice 14 days post-fracture. N = 6. Scale bars represent 50 µm. Box plots are depicted as interquartile range (=box), median (=line) and minimum to maximum values (=whiskers).

**Figure 7:**
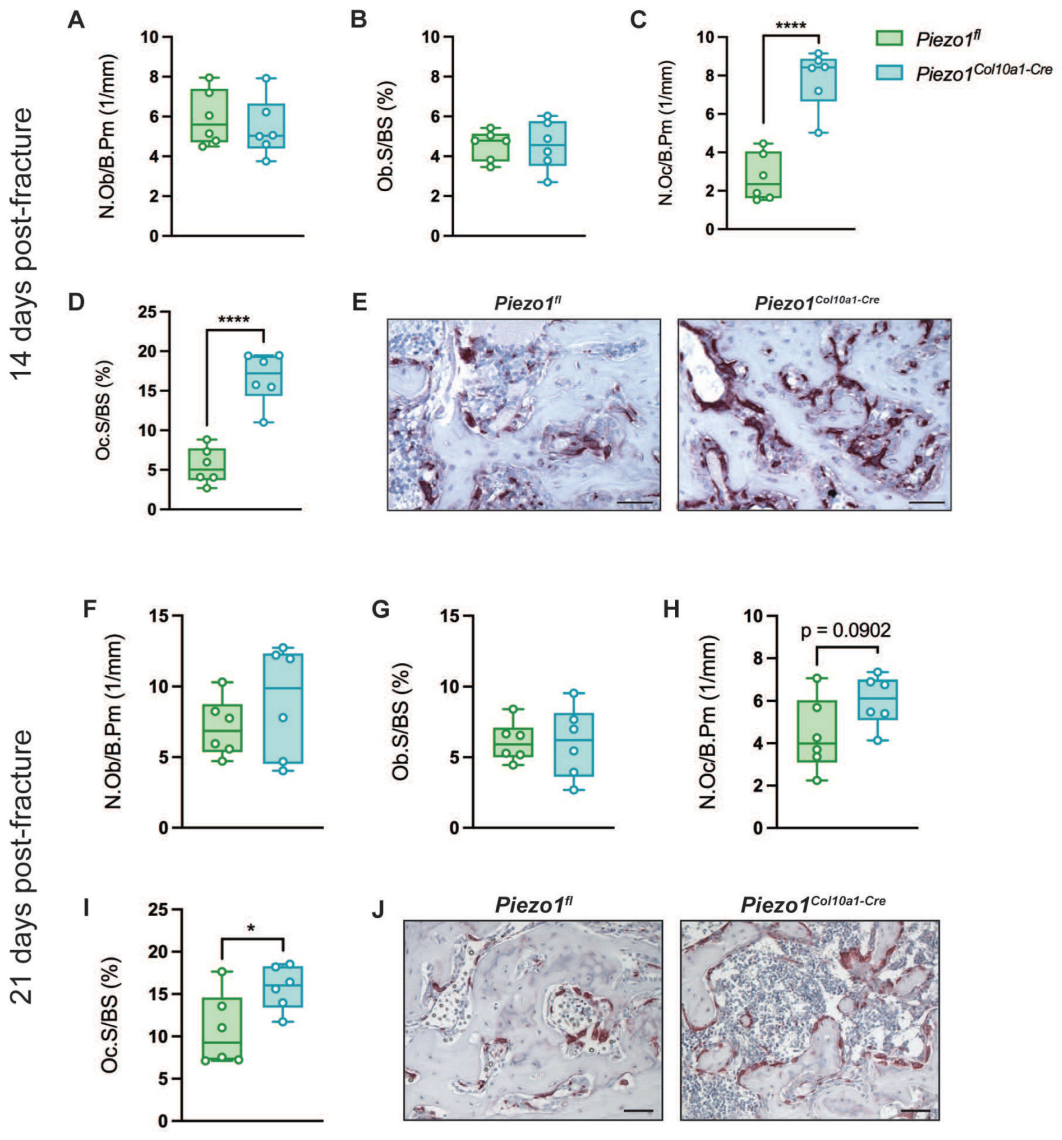
(A, F) N.Ob/B.Pm, (B, G) Ob.S/BS, (C, H) N.Oc/B.Pm, and (D, I) Oc.S/BS in the fracture callus of *Piezo1^fl^* and *Piezo1^Col10a1-Cre^* mice and (E, J) representative images of TRAP-stained fracture callus sections (A-E) at 14 days and (F-G) at 21 days post-fracture. N = 6, *p<0.05, ****p<0.0001. Scale bars represent 50 µm. Box plots are depicted as interquartile range (=box), median (=line) and minimum to maximum values (=whiskers).

**Figure 8:**
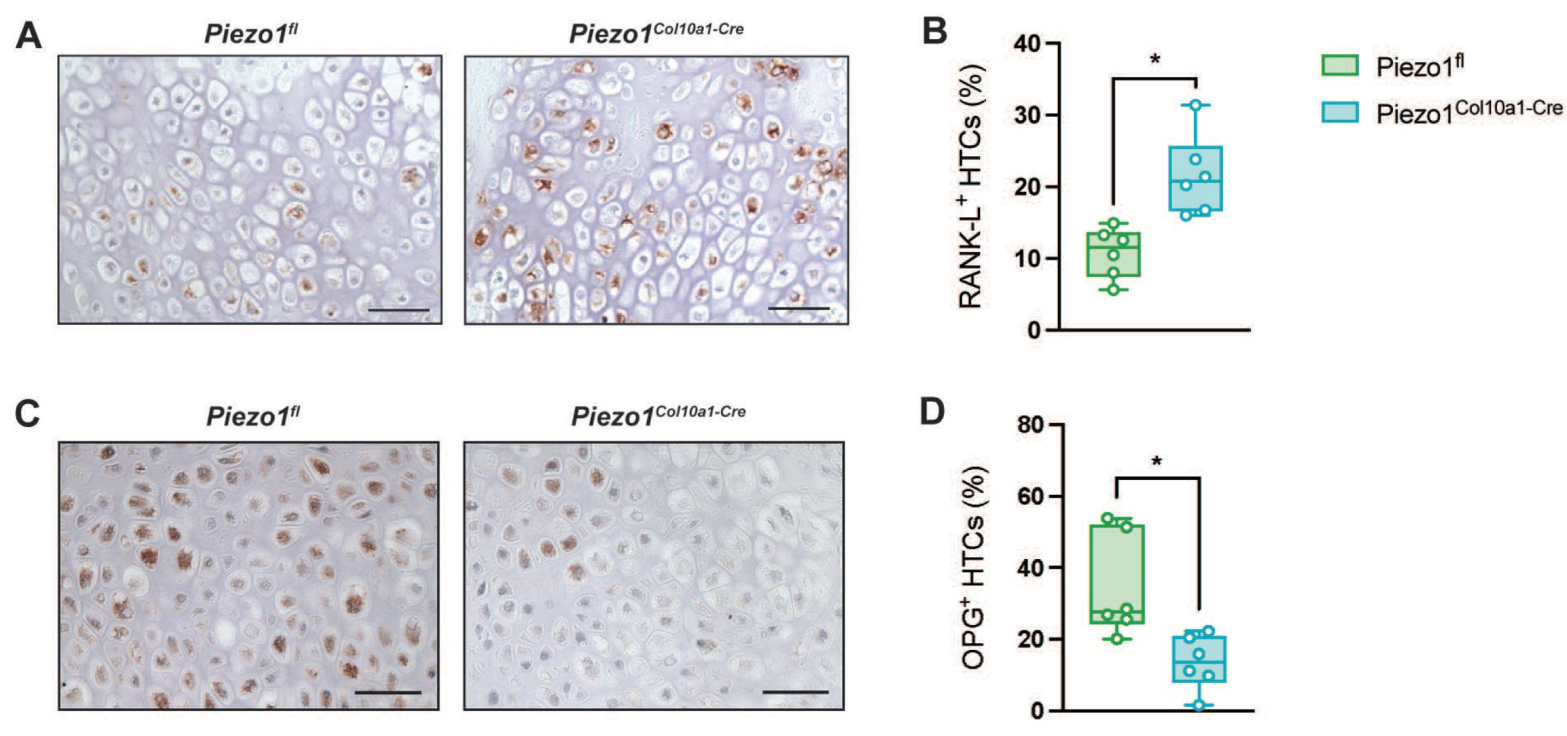
(A) Immunohistochemical staining of RANKL and (B) percentage of RANKL^+^ HTCs in the fracture callus of *Piezo1^fl^* and *Piezo1^Col10a1-Cre^* mice 14 days post-fracture and (C) immunohistochemical detection of osteoprotegerin (OPG) and (D) percentage of OPG^+^ cells in the fracture callus of *Piezo1^fl^* and *Piezo1^Col10a1-Cre^* mice 10 days post-fracture. Scale bars represent 50 µm. N = 6, *p<0.05. Box plots are depicted as interquartile range (=box), median (=line) and minimum to maximum values (=whiskers).

Consistent with the findings in the growth plate, Runx2 and Sox2 expression in hypertrophic chondrocytes within the fracture callus remained unchanged in *Piezo1^Col10a1-Cre^* mice (Fig. 6 A-D), as did both, TUNEL staining for chondrocyte apoptosis (Fig. 6 E, F) and osteoblast parameters (Fig. 7 A, B). By contrast, N.Oc/B.Pm (Fig. 7 C) and Oc.S/BS (Fig. 7 D, E) were significantly increased at day 14 and showed a non-significant trend towards higher values at 21 days post-fracture (p=0.0902) (Fig. 7 H-J). Furthermore, confirming the findings in the growth plate, RANKL expression was significantly upregulated in hypertrophic chondrocytes in *Piezo1^Col10a1-Cre^* mice (Fig. 8 A, B), whereas OPG expression was reduced in this cell type (Fig. 8 C, D).

**Figure 9:**
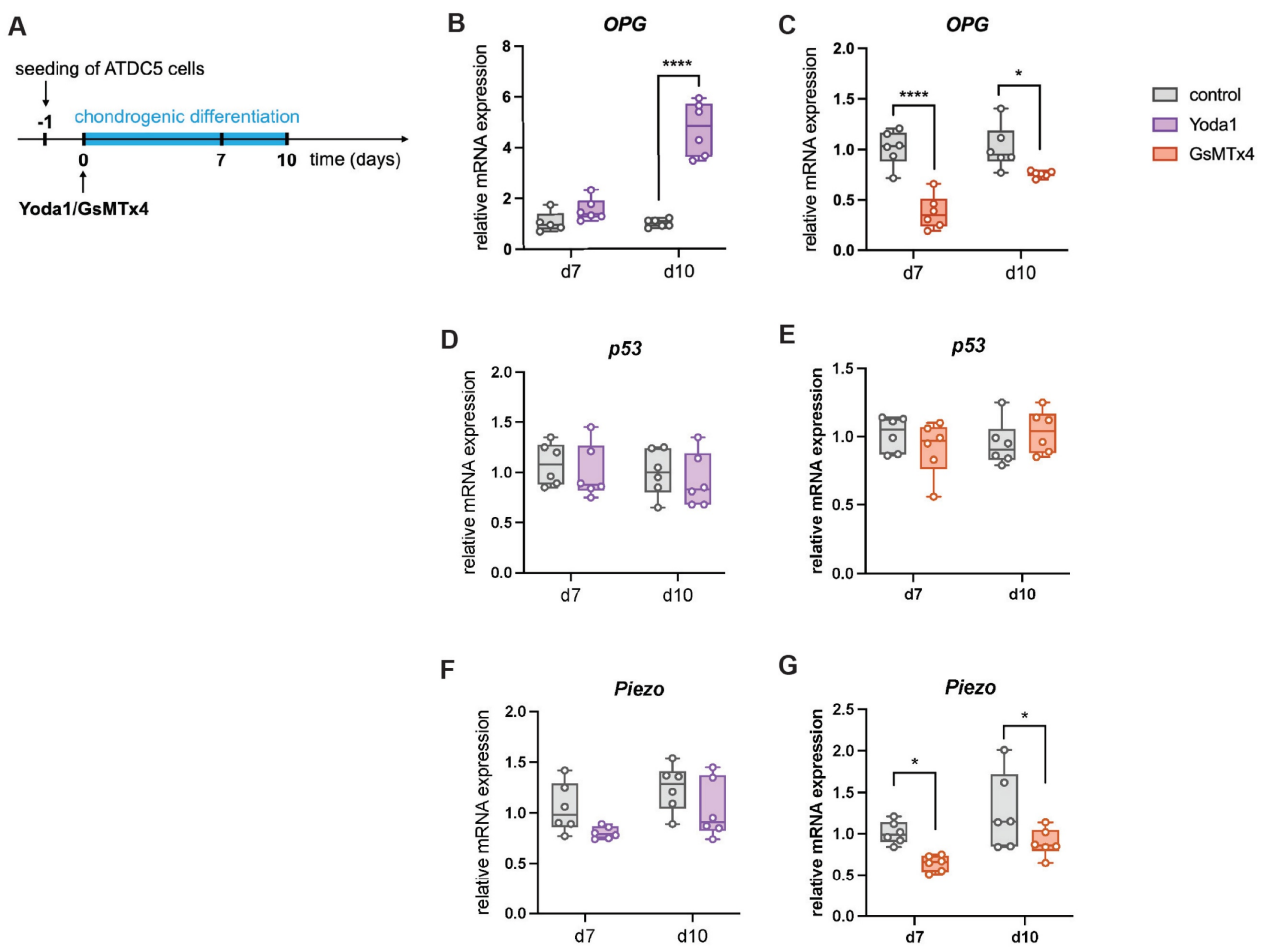
(A) Experimental setup to test the effect of Yoda1 and GsMTx4 on the gene expression of murine chondrogenic ATDC5 cells by the addition of the components to the chondrogenic differentiation medium. (B, C) Relative mRNA expression of *OPG* in ATDC5 cells treated with Yoda1 or GsMTx4. (D, E) Relative mRNA expression of *p53* in ATDC5 cells treated with Yoda1 or GsMTx4. (F, G) Relative mRNA expression of *Piezo1* in ATDC5 cells treated with Yoda1 or GsMTx4. N = 5-6, *p<0.05, **p<0.01, ***p<0.001, ****p<0.0001. Box plots are depicted as interquartile range (=box), median (=line) and minimum to maximum values (=whiskers).

**Figure 10:**
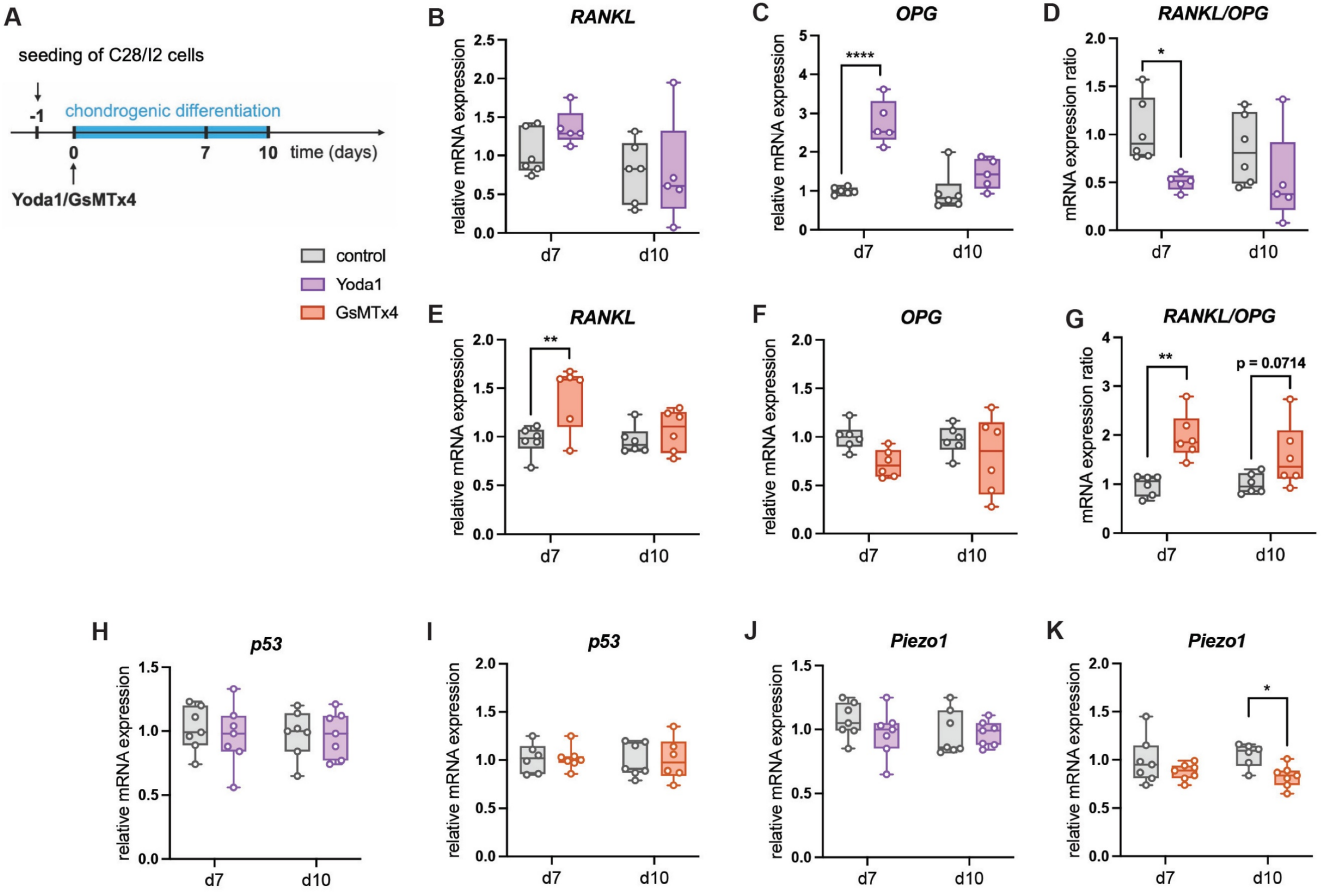
(A) Experimental setup to test the effect of Yoda1 and GsMTx4 on the gene expression of human chondrogenic C28/I2 cells, relative mRNA expression of (B, E) *RANKL*, (C, F), *OPG* (D, G) *RANKL/OPG* expression ratio, p53 (H, I) and Piezo1 (J, K) in C28/I2 cells treated with Yoda1 or GsMTx4. N = 5-6, *p<0.05, **p<0.01, ***p<0.001, ****p<0.0001. Box plots are depicted as interquartile range (=box), median (=line) and minimum to maximum values (=whiskers).

To mechanistically confirm the role of Piezo1 in the communication between chondrocytes and osteoclasts, differentiated murine chondrocytes were treated with the Piezo1 activator Yoda1 (38 or the ion channel antagonist GsMTx4 which antagonizes Piezo1 activity 39 (Fig. 9 A). Yoda1 induced OPG expression (Fig. 9 B), whereas GsMTx4 suppressed it (Fig. 9 C). P53 expression was unchanged under both treatments (Fig. 9 D, E), while GsMTx4 treatment significantly reduced expression of Piezo1 (Fig. 9 F, G). Given low RANKL expression in this murine cell line, experiments were repeated in human C28/I2 chondrocytes (Fig. 10 A). In this cell line, Yoda1 reduced the RANKL/OPG ratio (Fig. 10 D), whereas GsMTx4 increased it (Fig. 10 G). P53 expression was again unchanged (Fig. 10 H, I), while GsMTx4 treatment significantly reduced expression of Piezo1 at day 10 of differentiation (Fig. 10 J, K). Neither Yoda1 nor GsMTx4 treatment had an effect on in vitro chondrocyte-to-osteoblast transdifferentiation Fig. S5.

**Figure 11:**
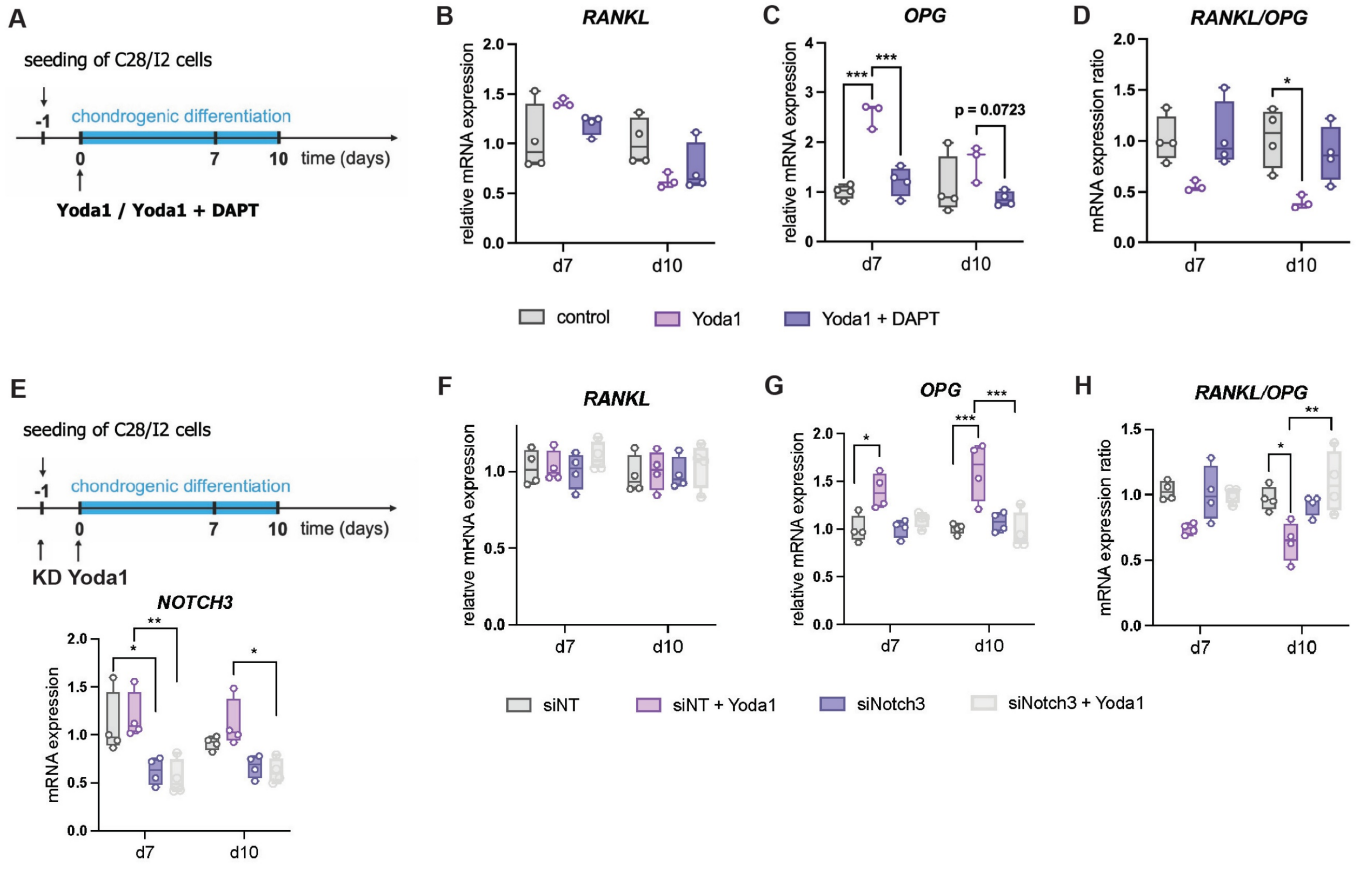
(A) Experimental setup of testing the effect of DAPT on Yoda1-treated C28/I2 cells, (B,C) *RANKL* and *OPG* expression and (D) *RANKL/OPG* expression ratio in cells treated with Yoda1 and Yoda1 + DAPT. (E) Experimental setup of testing the effect of siRNA-mediated Notch3 Knockdown (KD) on Yoda1-treated C28/I2 cells and knockdown efficiency, (F,G) *RANKL* and *OPG* expression and (H) *RANKL/OPG* expression ratio. N = 3-4, *p<0.05, **p<0.01, ***p<0.001, ****p<0.0001. Box plots are depicted as interquartile range (=box), median (=line) and minimum to maximum values (=whiskers).

To identify a potential signaling pathway involved, we examined the effect of the Notch inhibitor DAPT on Yoda1 treated C28/I2 cells (Fig. 11 A). DAPT significantly reduced Yoda1-induced OPG expression and showed a trend toward increasing the RANKL/OPG ratio in Yoda1-treated cells (Fig. 11 B-D). We next were interested if this effect is mediated by Notch3. Therefore, we further investigated effects of a siRNA-mediated downregulation of Notch3 on Yoda1 treated C28/I2 cells (Fig. 11 E). Again, RANKL expression was unaltered, while OPG expression was increased by Yoda1-treatment, but only in control cells transfected with non-targeting siRNA (siNT). Due to that, the RANKL/OPG ratio was also reduced by Yoda1, but only in siNT cells. Knockdown of Notch3 abolished the effects of Yoda1.

## Discussion

Our results demonstrated that Piezo1 in hypertrophic chondrocytes is a critical regulator of hypertrophic chondrocyte-osteoclast communication during endochondral bone formation in both the growth plate and the fracture callus during bone repair.

Previous studies, including our own, established that Piezo1 in differentiating chondrocytes is essential for endochondral ossification during bone development and fracture healing 22, 26-29. To specifically dissect the role of Piezo1 in hypertrophic chondrocytes, we generated a *Piezo1^Col10a1-Cre^* mouse model. Similar to *Piezo1^Col2a1-Cre^* mice 22, 26, *Piezo1^Col10a1-Cre^* mice exhibited low bone mass and spontaneous rib fractures. However, unlike *Piezo1^Col2a1-Cre^* mice 22, 26, they did not display impaired long bone growth or pelvic deformations. Moreover, growth plate thickness and the proportion of the hypertrophic zone were unchanged, indicating that Piezo1 deficiency in hypertrophic chondrocytes does not disrupt cartilage formation and maturation. Consistent with this, the formation of hypertrophic cartilage in the fracture callus was also unaffected. However, the markedly reduced trabecular bone mass and impaired callus maturation of *Piezo1^Col10a1-Cre^* mice underscore the importance of Piezo1 in hypertrophic chondrocytes in endochondral bone formation.

Given that hypertrophic chondrocytes can either undergo apoptosis or transdifferentiate into osteoblasts during endochondral ossification, we examined both processes. *Piezo1^Col10a1-Cre^* mice exhibited no differences in the expression of transdifferentiation markers in the growth plate or osteoblast numbers in the primary spongiosa compared to *Piezo1^fl^* controls, suggesting that the impaired bone formation is not due to defective chondrocyte-to-osteoblast transdifferentiation. This was also verified in an in vitro assay. These findings were also corroborated during fracture repair: neither transdifferentiation of hypertrophic chondrocytes nor osteoblast numbers within the fracture callus were altered 14 days post-fracture, and cartilage persistence at 21 days was not increased in *Piezo1^Col10a1-Cre^* mice. Chondrocyte apoptosis was likewise unchanged in *Piezo1^Col10a1-Cre^* mice compared to *Piezo1^fl^* controls.

Jia *et al*. previously reported that Piezo1-deficient chondrocytes in early stage of differentiation exhibit increased senescence, leading to defective endochondral ossification 27, a mechanism that may explain the impaired bone growth we previously observed in *Piezo1^Col2a1-Cre^* mice 22, 26. By contrast, *Piezo1^Col10a1-Cre^* mice displayed normal bone growth, suggesting that Piezo1 plays a pivotal role earlier in chondrogenesis rather than during transdifferentiation or apoptosis of hypertrophic chondrocytes. This conclusion was further supported by our finding that cartilage formation itself in the fracture callus was unaffected in *Piezo1^Col10a1-Cre^* mice. These results indicated that Piezo1 is indispensable in proliferative and differentiating chondrocytes, potentially through the regulation of chondrocyte senescence, while the subsequent transdifferentiation of hypertrophic chondrocytes into osteoblasts appears to be unaffected when Piezo1 is only deleted at later stages of chondrocyte hypertrophy. Interestingly, Zhang *et al*. 28 could demonstrate that loss of Piezo1 at an early stage in Col2a1-expressing chondrocytes indeed impairs chondrocytes-to-osteoblast transdifferentiation, indicating that the developmental stage at which Piezo1-knockout is introduced into chondrocytes is critical for priming these cells toward a differentiation-defective phenotype.

Instead, our data indicated that the low bone mass phenotype and impaired fracture healing observed in *Piezo1^Col10a1-Cre^* mice are driven by enhanced osteoclastogenesis and bone resorption, as evidenced by increased osteoclast numbers in the primary spongiosa and fracture callus. The increased osteoclast formation and activity appears to be mediated by altered RANKL and OPG expression, which we detected in the growth plate, primary spongiosa, and fracture callus of *Piezo1^Col10a1-Cre^* mice. RANKL was significantly upregulated in hypertrophic chondrocytes in the absence of *Piezo1*. Although OPG was not expressed in growth plate chondrocytes of both *Piezo1^fl^* and *Piezo1^Col10a1-Cre^* mice Fig. S3, its expression was markedly reduced in osteoblasts of the primary spongiosa. Given that hypertrophic chondrocytes have been shown to contribute to osteoblast populations through transdifferentiation during endochondral ossification 8, 9, it cannot be excluded that a subset of osteoblasts in the primary spongiosa of Piezo1^Col10a1^-Cre mice originates from the Col10a1 lineage and carries the Piezo1 deletion. Consequently, the reduced OPG expression observed in osteoblast-rich regions may reflect both direct lineage effects and altered signaling from hypertrophic chondrocytes. Future lineage-tracing studies will be required to distinguish between these mechanisms. Interestingly, RANKL expression in these osteoblasts was not altered by Piezo1 deletion, suggesting regulation of RANKL and OPG may differ depending on the cell type and cellular origin. Direct evidence for this would again require linage-tracing experiments to determine which osteoblasts in the primary spongiosa are derived from hypertrophic chondrocytes and to assess their specific expression patterns. Furthermore, co-staining of RANKL/OPG and hypertrophy markers like Col10a1 or Mmp13 and co-culture rescue experiments would strengthen our hypothesis that altered expression in hypertrophic chondrocytes would be the basis of the observed phenotype.

In the fracture callus, hypertrophic chondrocytes also appear to play a crucial role in osteoclast regulation: we observed robust expression of both RANKL and OPG in a large proportion of hypertrophic chondrocytes and marked differences between *Piezo1^Col10a1-Cre^* mice and *Piezo1^fl^* controls. The differing OPG expression patterns likely reflect the distinct physiological contexts of developmental endochondral ossification versus injury-induced repair. Although it has been shown that endochondral ossification during fracture healing mimics this process during development, these are still two distinct processes. In the steady-state growth plate, hypertrophic chondrocytes normally show minimal OPG expression, consistent with published literature describing OPG production predominantly by osteoblast-lineage cells in physiological bone remodeling 40. In contrast, fracture repair represents a highly dynamic environment characterized by inflammation, hypoxia, altered mechanical load, and recruitment of multiple cell lineages. These conditions are known to induce context-specific gene expression programs in chondrocytes during repair, including reactivation of genes not typically expressed during normal growth plate maturation. The increased OPG expression in hypertrophic chondrocytes of the fracture callus may therefore represent an injury-specific, reparative response rather than a developmental pattern.

Consistent with our *in vivo* findings, chemical activation of Piezo1 increased OPG expression in murine ATDC5 chondrogenic cells, whereas inhibition reduced it. Supporting, in human chondrogenic C28/I2 cells, Piezo1 activation reduced the RANKL/OPG expression ratio, whereas Piezo inhibition increased it, supporting that the elevated osteoclast formation and activity observed in *Piezo1^Col10a1-Cre^* mice is mediated by the Piezo1-mediated RANKL and OPG expression in hypertrophic chondrocytes.

Enhanced osteoclastogenesis has previously been implicated in low bone mass in *Piezo1*-deficient osteoblast lineage mice 25; however, that study did not detect differences in RANKL or OPG expression. In agreement with our data, Piezo1 activation has been shown to inhibit RANKL and promote OPG expression in osteocytes *in vitro* 41. While osteocytes are recognized as the primary regulators of osteoclastogenesis through RANKL production 42, 43, chondrocytes have also been reported to influence bone resorption by expressing RANKL and OPG 44. Our study provides the first evidence that Piezo1 in hypertrophic chondrocytes regulates osteoclastogenesis via modulation of RANKL and OPG expression during bone development and fracture repair.

To identify potential signaling pathways mediating Piezo1-dependent regulation of osteoclastogenesis, we screened the literature for pathways known to influence the RANKL/OPG axis. The reversal of Yoda1's effects by the Notch inhibitor DAPT in C28/I2 cells indicates that Piezo1 modulates OPG expression by Notch signaling. This finding aligns with previous work from Liu *et al*., which demonstrated that Piezo1-induced OPG upregulation and RANKL inhibition in osteocytes is mediated by NOTCH3 41. We also found that siRNA-mediated knockdown of Notch3 attenuated the effects of Yoda1, suggesting that Notch3 signaling may contribute to mediating Piezo1-dependent regulation in chondrocytes. However, these data do not establish a direct causal relationship in vivo. In addition to Notch signaling, other pathways, including canonical Wnt signaling, which has been implicated in hypertrophic chondrocyte-driven osteoclastogenesis 45, may also be involved. Taken together, our findings, in combination with previous reports, are consistent with a role for Notch signaling in Piezo1-associated regulation of osteoclastogenesis, although further studies are required to define the precise mechanistic interactions.

Together, our results indicated that Piezo1 in hypertrophic chondrocytes regulates osteoclastogenesis by suppressing RANKL and inducing OPG expression, thereby contributing to bone formation during endochondral ossification under physiologic and pathologic conditions.

## Supplementary Material

Supplementary figures and table.

## Figures and Tables

**Table 1:** CTX serum levels in pg/ml in male and female *Piezo1^Col10a1-Cre^* and *Piezo1^fl^* at the age of 12 weeks.

sex	*Piezo1^fl^*	*Piezo1^Col10a1-Cre^*
male	16.4 ± 14.0	55.6 ± 36.1*
female	5.3 ± 33.7	22.5 ± 10.3*

## Data Availability

The authors declare that all primary data points are displayed in the manuscript and its corresponding Supplementary Information files. All raw data supporting the findings of this study are available from the corresponding author upon request.
