# Supplementary material for: Piezo1 in hypertrophic chondrocytes regulates osteoclastogenesis in endochondral ossification
Source: Int J Biol Sci. 2026 Apr 23;22(9):4863–77. doi: 10.7150/ijbs.126065 (PMC13182562; doi:10.7150/ijbs.126065)
Supplement: Supplementary file 1 — Supplementary figures and table. [file ijbsv22p4863s1.pdf]

Supporting information

Piezo1 in hypertrophic chondrocytes regulates osteoclastogenesis during bone development and fracture healing

Miriam E.A. Tschaffon-Müller, Astrid Schoppa, Franziska Eckl, Laura J. Brylka, Melanie Haffner-Luntzer, Timur A. Yorgan, Sandra Dieterich, Christoph Kölbl, Michael Amling, Thorsten Schinke, Anita Ignatius

Corresponding author: Prof. Dr. Anita Ignatius
Email: anita.ignatius@uni-ulm.de

This file includes:
Table S1
Figures S1-S5

Supplementary Tables

Table S1. Primer pairs used for quantitative real-time PCRs in murine ATDC5 cells and human C28/I2 cells.

Gene	forward primer sequence (5' to 3')	reverse primer sequence (3' to 5')
murine *GAPDH*	ACCCAGAAGACTGTGGATGG	GGATGCAGGGATGATGTTCT
murine *OPG*	CTGCCTGGGAAGAAGATCAG	TTGTGAAGCTGTGCAGGAAC
human *GAPDH*	GAAGGTGAAGGTCGGAGTC	GAAGATGGGATGGGATTTC
human *RANKL*	CCAGCATCAAAATCCCAAGT	CCCCAAAGTATGTTGCATCCTG
human *OPG*	AGGAAATGCAACCAACGACA	TACTTTGGTGCCAGGCAAAT
murine p53	GGAAATTTGTATCCCGAGTATCTG	GTCTTCCAGTGTGATGATGGTAA
murine Piezo1	CTCCGACTTCCTCGAGTGGT	CTAGGTGGGCTGACCTTGTC

Supplementary Figures

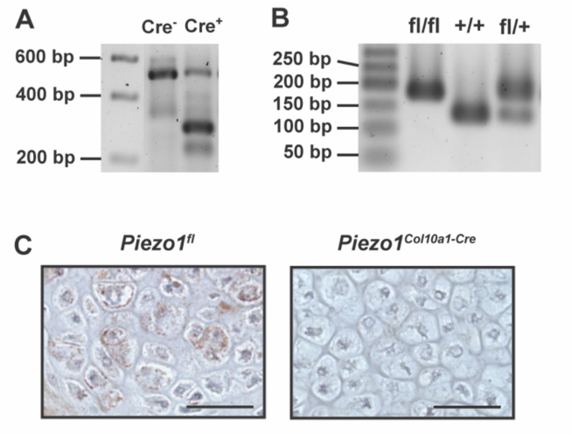

Figure S1. Gel documentation of (A) the wildtype (500 bp) and *Col10a1-Cre* PCR product (300 bp) and (B) the floxed (fl, 200 bp) and wildtype (+, 150 bp) *Piezo1* allele PCR product. (C) Immunohistochemical detection of the Piezo1 extracellular domain on fracture callus sections of *Piezo1*^{fl} and *Piezo1*$^{Col10a1\text{-}Cre}$ mice. Scale bars represent 50 µm.

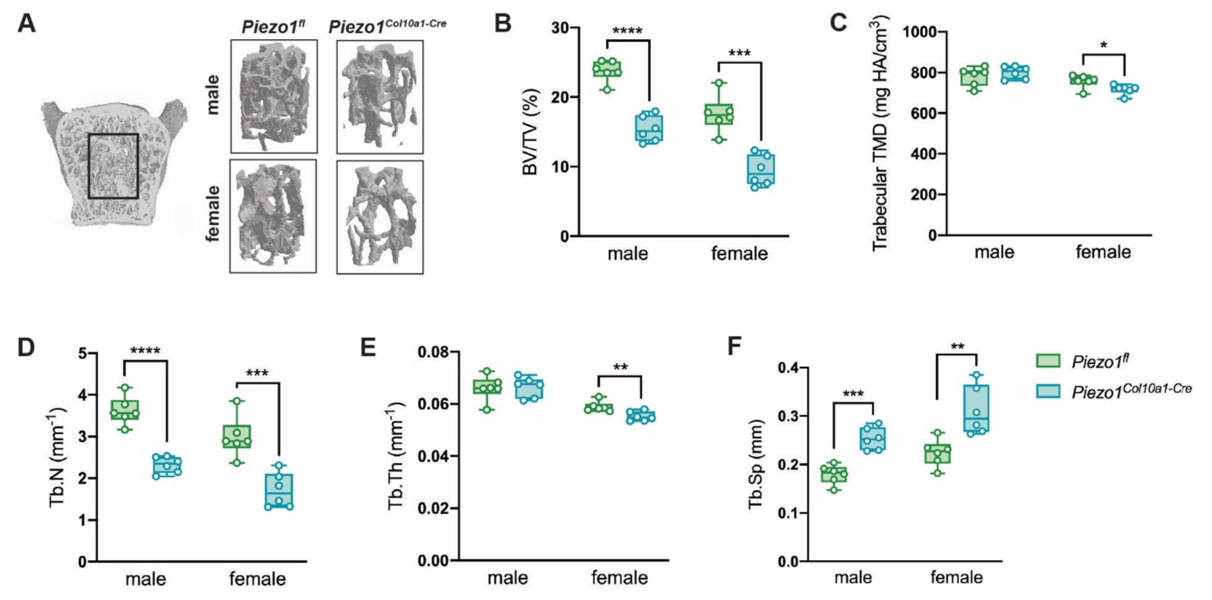

Figure S2. (A) Representative images of analyzed ROIs in lumbar vertebra L4, (B) bone volume/tissue volume ratio (BV/TV), (C) trabecular tissue mineral density (TMD), (D) trabecular number (Tb.N), (E) trabecular thickness (Tb.Th) and (F) trabecular separation (Tb.Sp) of spines of 12-week-old male and female *Piezo1*^{fl} and *Piezo1*$^{Col10a1\text{-}Cre}$ mice. N = 6, *p<0.05, **p<0.01, ***p<0.001, ****p<0.0001.

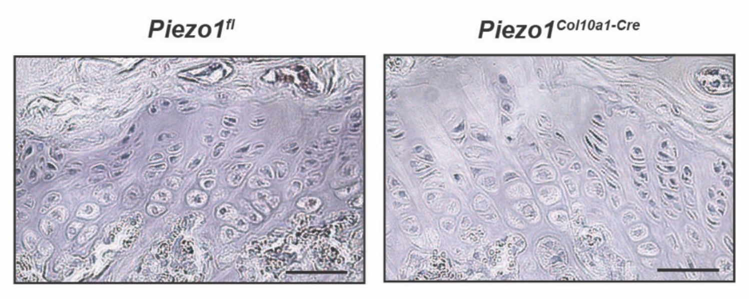

Figure S3. Immunohistochemical detection of OPG in growth plates of *Piezo1*^{fl} and *Piezo1*$^{Col10a1\text{-}Cre}$ mice. Scale bars represent 50 µm.

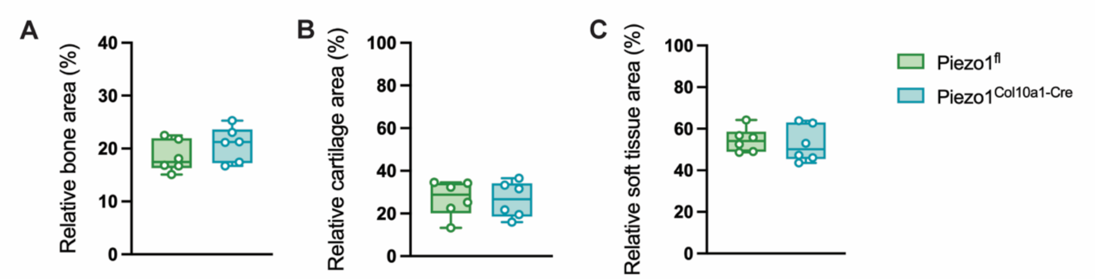

Figure S4. Relative (A) bone, (B) cartilage and (C) soft tissue area in fracture calli of *Piezo1*^{fl} and *Piezo1*$^{Col10a1\text{-}Cre}$ mice 14 days post-fracture. N = 6.

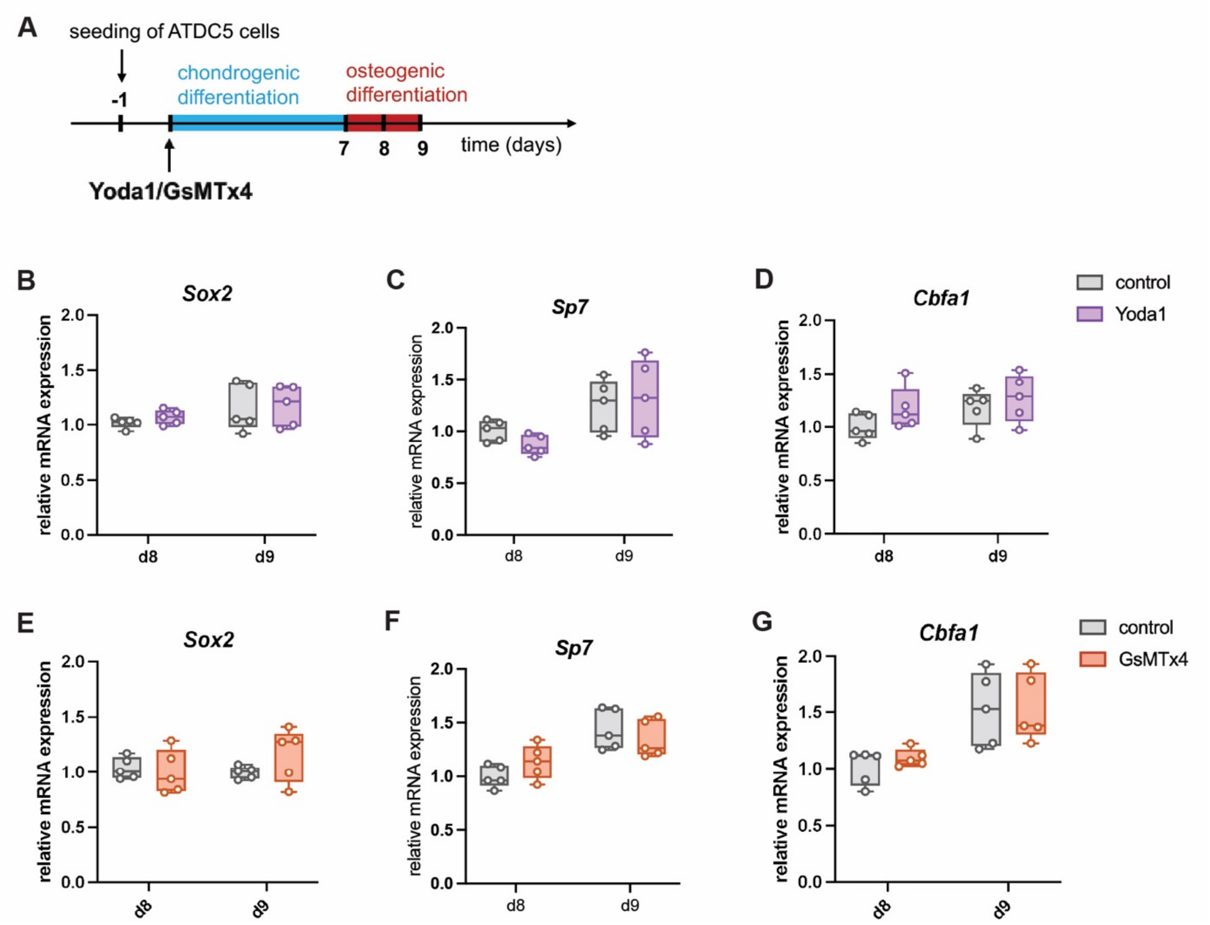

Figure S5. (A) Experimental setup of testing the effect of Yoda1 and GsMTx4 on the transdifferentiation of chondrogenic ATDC5 cells. Relative mRNA expression of (B, E) *Sox2,* (C, F) *Sp7*, (D, G) *Cbfa1* in transdifferentiating ATDC5 cells treated with Yoda1 or GsMTx4. N = 5, *p<0.05, **p<0.01, ***p<0.001, ****p<0.0001.

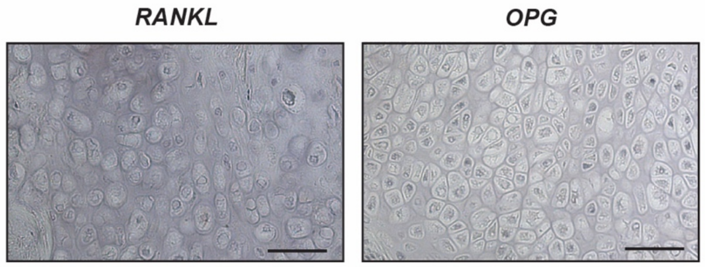

Figure S6. Representative negative control stainings for RANKL and OPG in the fracture callus.
